# Methods matter: Exploring how expectations influence common actions

**DOI:** 10.1016/j.isci.2024.109076

**Published:** 2024-02-01

**Authors:** Andrea Ghiani, David Mann, Eli Brenner

**Affiliations:** 1 Department of Human Movement Sciences, Amsterdam Movement Sciences and Institute of Brain and Behaviour Amsterdam, Vrije Universiteit Amsterdam, Amsterdam, the Netherlands

**Keywords:** Behavioral neuroscience, Social sciences, Research methodology social sciences

## Abstract

Behavior in controlled laboratory studies is not always representative of what people do in daily life. This has prompted a recent shift toward conducting studies in natural settings. We wondered whether expectations raised by how the task is presented should also be considered. To find out, we studied gaze when walking down and up a staircase. Gaze was often directed at steps before stepping on them, but most participants did not look at every step. Importantly, participants fixated more steps and looked around less when asked to navigate the staircase than when navigating the same staircase but asked to walk outside. Presumably, expecting the staircase to be important made participants direct their gaze at more steps, despite the identical requirements when on the staircase. This illustrates that behavior can be influenced by expectations, such as expectations resulting from task instructions, even when studies are conducted in natural settings.

## Highlights

• Experimental tasks are often designed to sample real-world behavior
• We studied gaze when climbing a staircase
• Participants who expected the staircase was important fixated more steps
• Subtle changes in how tasks are presented can change how the task is performed

## Graphical abstract

**Figure:**
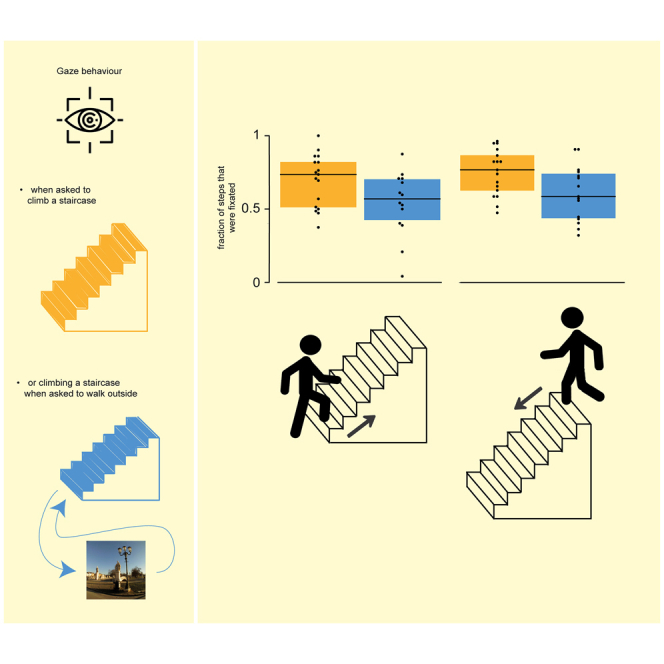

## Introduction

Research in experimental psychology has made a lot of progress by studying behavior in simple, well-controlled environments. However, results obtained in this manner do not always generalize to similar situations in daily life.^1^^,^^2^^,^^3^^,^^4^^,^^5^ Apparently, behavior in simple laboratory environments can differ from that in the complex situations encountered in the natural environment.^2^^,^^6^^,^^7^ For instance, studies on social attention find that people spend much more time directing their gaze toward faces presented on a screen than they do when required to make face-to-face contact.^8^^,^^9^^,^^10^^,^^11^ Such findings have encouraged psychological researchers to conduct their experiments in environments that are more representative of those in which the behavior under study usually takes place. Many examples of this trend can be found in studies of social cognition,^1^^,^^11^^,^^12^^,^^13^ education,^14^ eye movements,^15^^,^^16^^,^^17^^,^^18^ and memory.^19^^,^^20^ But is conducting the study under more natural settings enough to guarantee generalizability?

The experimental situation obviously creates expectations that can influence participants’ behavior, irrespective of the setting. For instance, when considering gaze, if people are simply instructed to look at a scene their gaze is largely driven by image features,^21^^,^^22^^,^^23^^,^^24^^,^^25^^,^^26^ but if they are explicitly asked to make certain judgments about the scene then their gaze is directed toward information that they expect to be relevant for that judgment.^20^^,^^27^^,^^28^^,^^29^^,^^30^ Gaze allocation is usually best explained by how relevant regions are expected to be for the task at each instant.^16^^,^^18^^,^^31^^,^^32^^,^^33^^,^^34^ Thus, it might be reasonable to expect that only the task and the environment matter, so that studying gaze in a natural setting is enough.

When asking participants to perform certain tasks in scientific experiments, we are not only informing them about what they need to do but also indirectly introducing expectations about the relevance of various components of the task. Doing so might also influence behavior. For instance, when asked to perform a version of the “‘running an errand” task where participants had to memorize a list of items and plan the shortest route to find these items in an imaginary city map,^35^ elderly people performed differently depending on the precise task instructions. Under regular conditions, elderly adults had similar performance to younger adults. However, when the instructions explicitly emphasized the memory component of the task, the elderly participants performed worse than the young adults.^36^ Thus, the instructions influenced participants’ expectations about their performance on the task, subsequently influencing performance itself. Similarly, the *framing effect* demonstrates that participants make different choices for equivalent options when instructions are formulated in different ways.^37^ For example, when asked to choose between treatments for a deadly disease, participants made different choices when treatments were framed positively (in terms of people saved) than when they were framed negatively (in terms of people dying). Thus, it is evident that expectations generated by task instructions can affect people’s behavior.^36^^,^^37^^,^^38^ However, these examples involved tasks with complex cognitive components that one might expect to be prone to contextual influences. Here, we wondered whether expectations might also influence the performance of simple, well-trained behavior in a natural setting.

**Figure 1:**
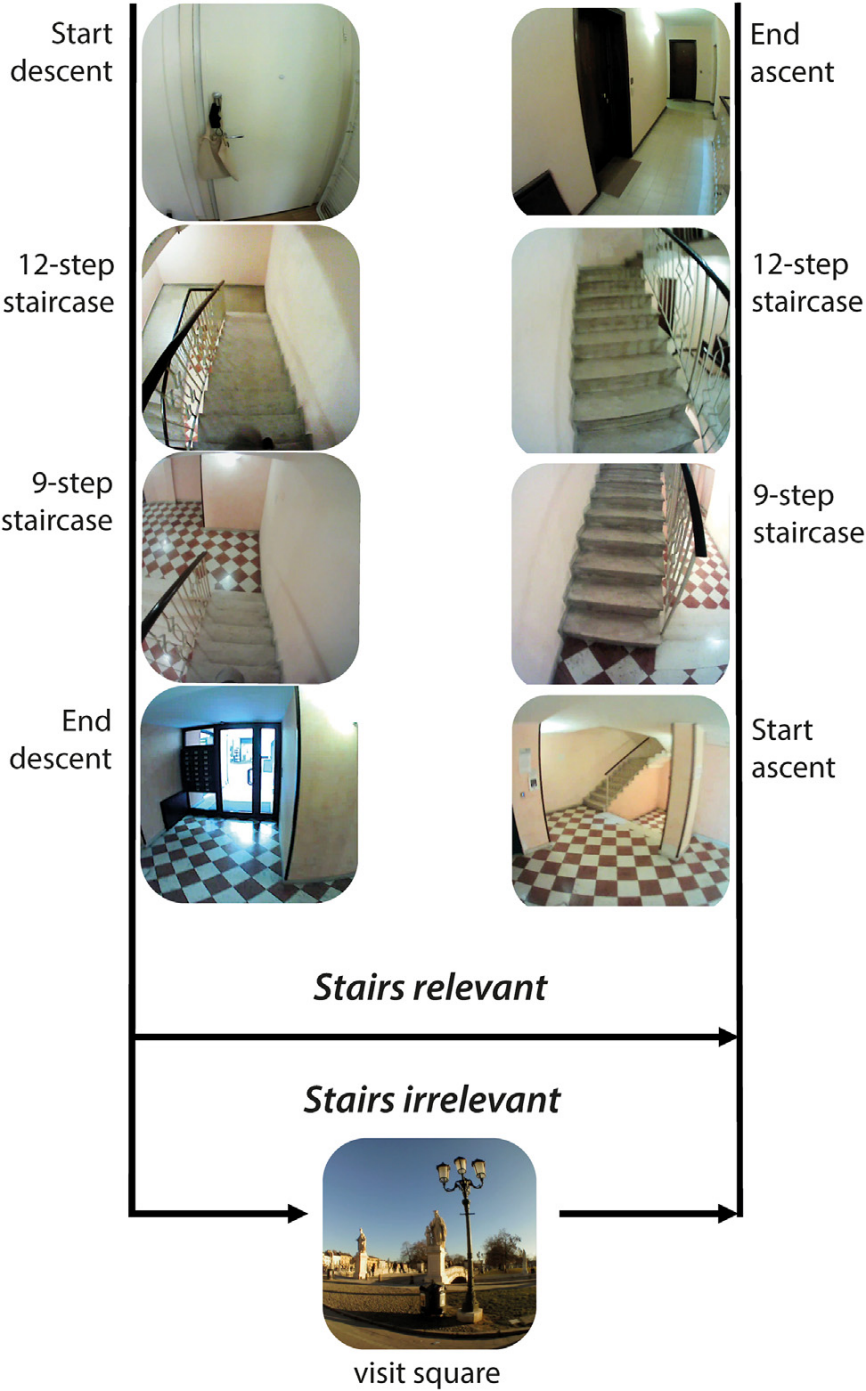
Illustration of the task for the two groups Critical parts of the participants’ trajectories in the *Stairs-relevant* and *Stairs-irrelevant* groups. The *Stairs-relevant* group walked down the 12-step staircase and then down the 9-step staircase (in addition to three short staircases that were not considered for the analysis). They reached the entrance door of the building (End descent), and then immediately returned (Start ascent). Once they had walked up both staircases the trial ended (End ascent). The structure of a trial for the *Stairs-irrelevant* group was the same, but when reaching the entrance of the building, participants walked out of the building to a nearby square and observed the statues there for 2–3 min before returning and ascending the stairs.

The behavior that we chose to study was how people direct their gaze when navigating staircases. We chose this behavior because it clearly relies on visual information, but people do not direct their gaze at every step, with there being considerable variability in the number of steps they fixate.^17^ We examined whether expectations influence such gaze behavior. Participants were divided into two groups (Figure 1). Participants in the *Stairs-relevant* group were explicitly asked to walk down a flight of stairs and then walk back up again. Those in the *Stairs-irrelevant* group were asked to walk to a nearby square, examine the statues at the square, and then walk back. They had to navigate the same staircase to do so, but the instructions contained no mention of the staircase. We hypothesized that participants in the *Stairs-relevant* group would expect stair navigation to be more important, and therefore would direct their gaze toward more steps than would participants of the *Stairs-irrelevant* group.

## Results

**Figure 2:**
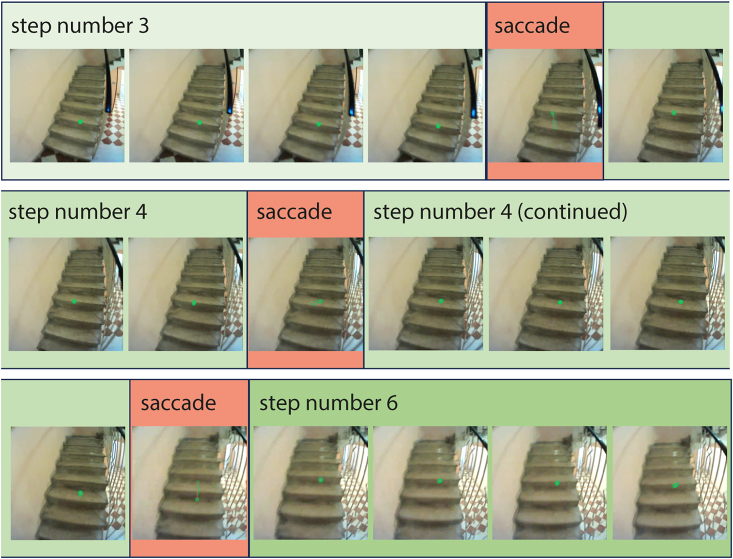
Illustration of the manual coding Manual coding was conducted in order to identify the fixated structures of interest. The images are consecutive frames from the video data. Red rectangles indicate that the image contains a saccade, as automatically detected with a custom-built script (see STAR Methods). Different shades of green indicate that a different step is fixated. The number indicates which step. In this example, a saccade was made within step number 4, so both fixations have the same shade of green (i.e., multiple fixations made within the same step were merged into one label). If gaze was not on a step, it was labeled as elsewhere, irrespective of its exact position.

Participants in both groups descended and ascended the same staircases. We manually coded each frame when they were on the staircase (see Figure 2) to identify the fixated steps and instances when gaze was directed elsewhere. We used this to determine the gaze sequence across steps and the number of fixated steps. We also determined the average saccade amplitude and the total time spent on the staircase (see STAR Methods for details). We compared these measures across the two groups.

### Gaze sequence and fraction of fixated steps

**Figure 3:**
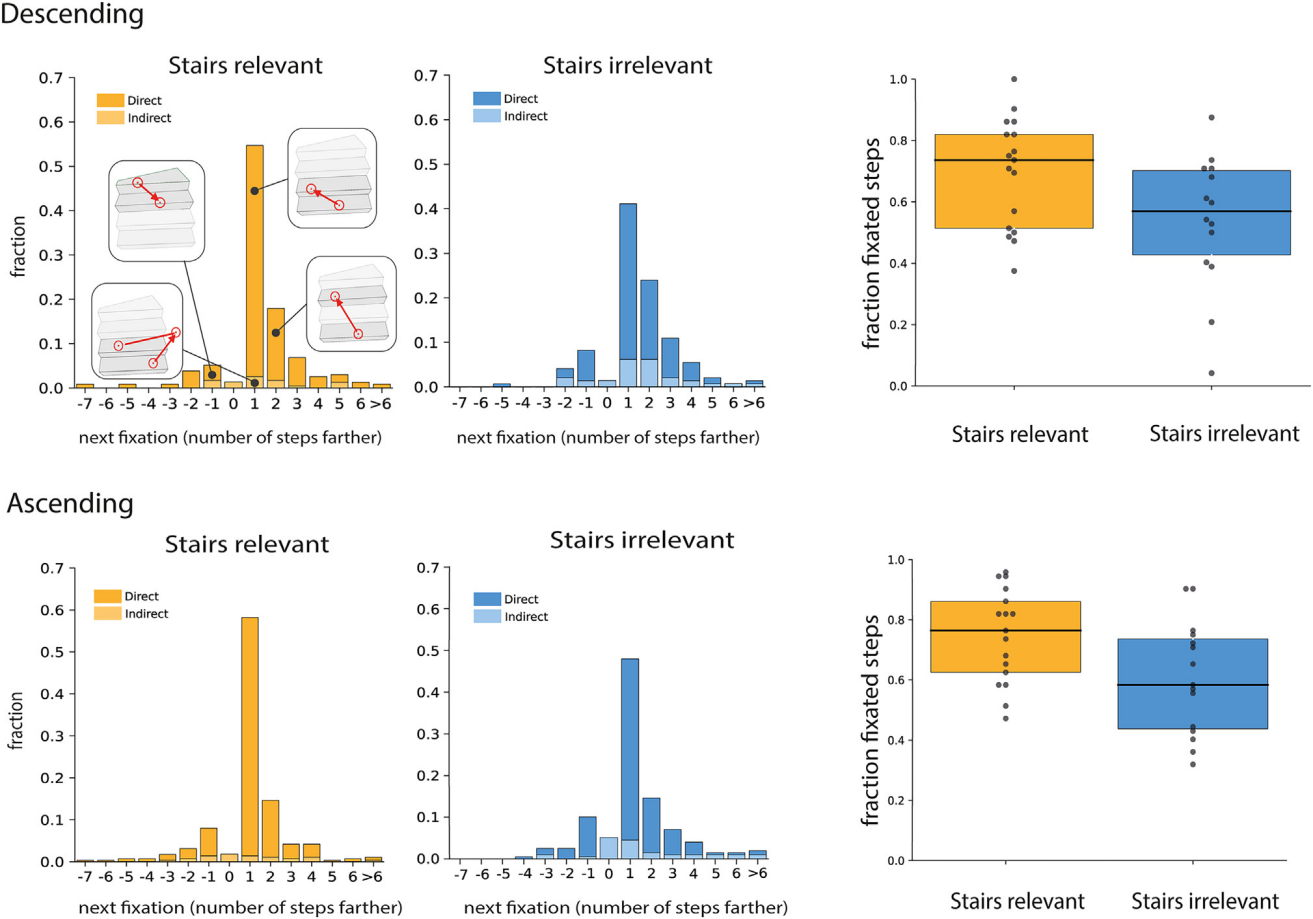
Frequency distribution of gaze sequences across steps and fraction of fixated steps Frequency distribution of how many steps farther participants looked on the subsequent fixation when descending (upper left panels) and ascending (bottom left panels) staircases, separately for the *Stairs-relevant* group (yellow) and *Stairs-irrelevant* group (blue). *Direct* distributions (dark colors) show how many steps farther the participants looked when consecutive fixations were both on steps. *Indirect* distributions (light colors) show how many steps farther they looked after looking elsewhere. Right panels: fraction of steps that were fixated in the *Stairs-relevant* and *Stairs-irrelevant* groups when descending (upper right panel) and ascending (bottom right panel). Dots represent individual participants. Boxplots show the median and intra-quartile range.

Gaze mainly shifted from one step to the next, both when ascending and descending the staircases, but it could also shift two or more steps ahead or one or more steps backwards. The frequency distribution of gaze shifts between sequentially fixated steps (Figure 3, left panels) suggests that participants in the *Stairs-relevant* group made more fixations across successive steps, as indicated by the higher peak at the “+1” step. Those participants also made fewer gaze shifts away from the steps and back (*Indirect*), both when descending (9% rather than 23% of gaze shifts) and ascending (9% rather than 18% of gaze shifts). Critically, participants in the *Stairs-relevant* group fixated almost 80% of the steps (Figure 3, right panels) whereas those in the *Stairs-irrelevant* group only fixated about 60% of the steps (similar to the value found in a previous study with no emphasis on the staircase^17^). An analysis of variance (ANOVA) revealed a significant main effect of Group (*Stairs-relevant* group vs. *Stairs-irrelevant* group) (F(1,29) = 5.50; p = 0.03, η_p_^2^ = 0.16), a significant main effect of Direction (Ascending vs. Descending) (F(1,29) = 5.47; p = 0.03, η_p_^2^ = 0.16), and no significant interaction (F(1,29) = 0.43; p = 0.52, η_p_^2^ = 0.01).

### Saccade directions and amplitudes

**Figure 4:**
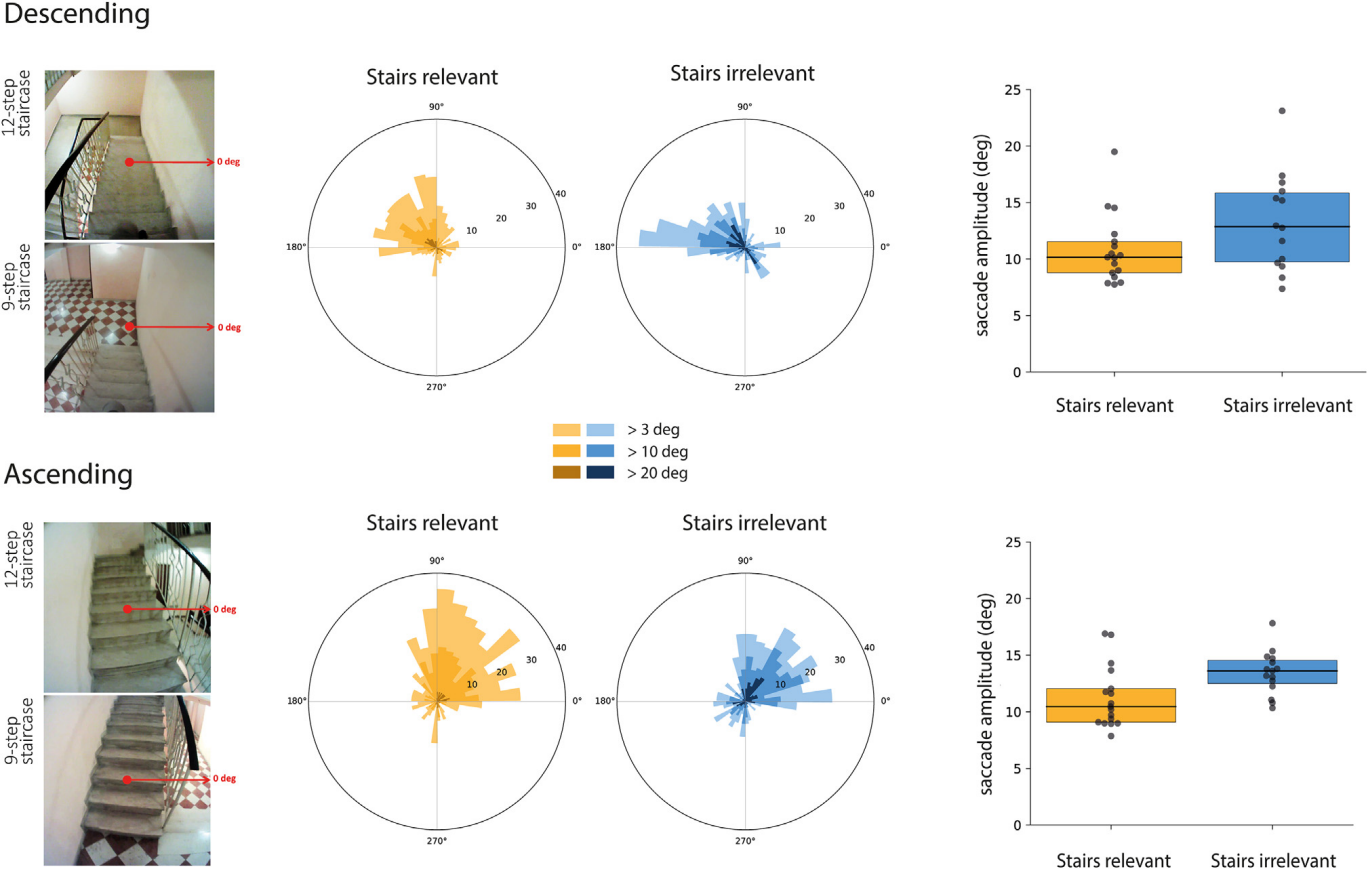
Distribution of saccadic directions and saccade amplitudes Distribution of saccadic directions (central panels) and mean saccade amplitudes (right panels) for the *Stairs-relevant* group (yellow) and *Stairs-irrelevant* group (blue) when descending (upper panels) and ascending (bottom panels). The pictures on the left show single frames from the scene video indicating the head-fixed 0-degree direction of the coordinate system used to define saccade directions. The shading in the central panels indicates the three minimal saccade amplitudes that we considered. Directions were grouped into 40 bins of 9° each.

Saccades were biased toward the open side of the staircase (away from the wall; Figure 4, left panels) in both groups: to the left when descending and to the right when ascending (Figure 4, central panels). This behavior was more pronounced for the *Stairs-irrelevant* group. Participants from the *Stairs-relevant* group tended to direct more saccades in the direction toward which they were moving (i.e., further along the staircase—upwards in the central panels of Figure 4). An ANOVA on the average saccadic amplitudes (right panels in Figure 4) revealed a main effect of Group (F(1,29) = 5.63, p = 0.02, η_p_^2^ = 0.16), with smaller saccades for the *Stairs-relevant* group. The main effect of Direction (F(1,29) = 0.23, p = 0.63, η_p_^2^ = 0.008) and the interaction (F(1,29) = 0.09; p = 0.76, η_p_^2^ = 0.003) were not significant.

### Time on stairs

**Figure 5:**
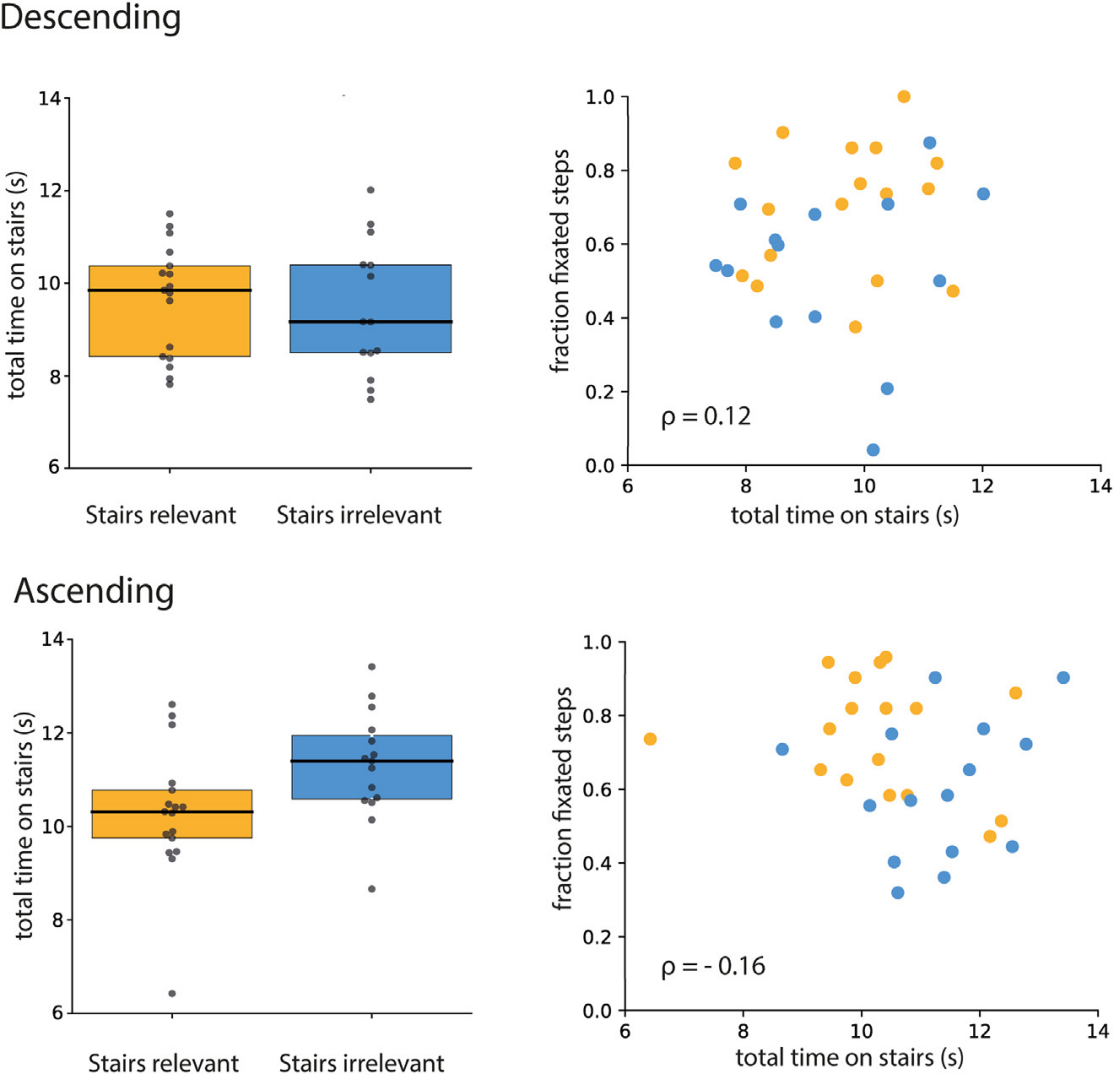
Time on stairs and correlation with the fraction of fixated steps Time spent on the staircase by the *Stairs-relevant* and *Stairs-irrelevant* groups, both when descending and ascending (left panels), and correlation between how much time individual participants spent on the staircase and the fraction of steps they fixated (right panels). Each dot represents a single participant. In the right panels, yellow dots represent participants from the *Stairs-relevant* group and blue dots represent participants from the *Stairs-irrelevant* group. Pearson correlation coefficients are computed across both groups, separately for ascending and descending.

To check whether the higher fraction of fixated steps with smaller saccades in the *Stairs-relevant* group could be explained by a difference in walking speed between the two groups, we computed the total time that each participant spent navigating the staircases. An ANOVA revealed no significant main effect of Group (F(1,29) = 1.03, p = 0.32, η_p_^2^ = 0.03). The main effect of Direction was significant (F(1,29) = 39.29, p < 0.001, η_p_^2^ = 0.58) as was the interaction (F(1,29) = 10.39; p = 0.003, η_p_^2^ = 0.26). People took more time when ascending, especially in the *Stairs-irrelevant* group (Figure 5, left panels). However, there was no correlation between the total time spent on the stairs and the fraction of fixated steps, showing that the time spent on the staircase does not determine the fraction of steps that are fixated in any simple manner (Figure 5, right panels).

## Discussion

We found that expectations raised by the way the task is presented influence behavior on an everyday task. Thus, conducting studies within natural settings is not enough to guarantee that the findings will also apply to daily life behavior. Providing task instructions that reveal the behavior of interest is common practice in psychological research, so this is an issue that needs to be taken into consideration, even when studying simple, well-trained behavior such as gaze allocation during stair navigation.

In most earlier studies of stair climbing, it was completely obvious that gaze on the stairs was the topic of investigation. In a study in which this was not the case, people fixated fewer steps than usually reported, but this was attributed to the experimental setting being more natural and the staircases being steeper.^17^ In the current study, we always used the same uncluttered flight of stairs. We compared performance after receiving instructions that either revealed or concealed the relevance of the staircase. We found that participants fixated more steps (Figure 3) and made fewer saccades away from the staircase (Figure 4) when the instructions revealed that the staircase was relevant. When the instructions did not reveal that the staircase was relevant, participants may have been more concerned with where they were heading and therefore have looked around more.

We attribute all the differences that we found between the two groups to the way the task was presented. However, the *Stairs-irrelevant* group not only received different instructions but also walked for an additional 10 min before ascending the staircase. We do not think this mattered much, because we see very similar trends when descending and ascending the staircases. Both groups started by descending the staircases, so the differences in the number of fixated steps and saccade amplitudes when descending the staircases cannot be attributed to the additional walking. The additional 10 min spent outside by the *Stairs-irrelevant* group might have made them less conscious of the eye tracker. It is probably also responsible for them walking up the stairs more slowly, as evidenced by a longer total time spent ascending the staircases (Figure 5). However, despite spending more time on the staircase, participants in this group tended to fixate fewer steps when walking up the stairs, just as they did when walking down (Figure 3). Thus, walking more slowly does not automatically lead to more steps being fixated just because there is more time to look at them. This is supported by the absence of a clear correlation between the time on the staircase and the fraction of fixated steps (Figure 5). Thus, it is probably justified to attribute the observed differences in gaze to how the task was presented.

Our findings are in line with earlier research showing that the experimental setting or task affects where humans look. For example, gaze behavior is different when experienced goalkeepers are asked to indicate the direction of a penalty shot verbally, than when they are asked to do so with an actual interceptive action.^39^ Gaze behavior is also different when people explore a real environment than when they explore a video of the same environment.^40^ As such, our findings contribute to the doubts about the extent to which the results of experimental studies inform us about behavior in daily life.^2^^,^^7^ We have built on this by showing that task instructions influence behavior by creating expectations about the relevance of certain aspects of the task, as is the case when task instructions activate stereotypes such as that evoked when elderly people are primed to focus on memory.^36^ It is well established that presenting a task in a different manner can influence people’s conscious or subconscious decisions.^37^ Here, we show a similar influence on the control of common actions in daily life. Although people are generally not conscious of their gaze being guided toward relevant structures when performing such actions, we find a bias toward what participants consider to be task-relevant structures. This bias emerges even though participants managed to navigate the staircase irrespective of the number of steps they viewed. This suggests that the way a task is presented has to be taken into consideration when studying daily life behavior. The behavior observed in the *Stairs-irrelevant* group is probably more similar to that in daily life than that in the *Stairs-relevant* group, because participants use the staircase to reach a destination, which is the usual purpose of walking on stairs, rather than as a goal in itself. Embedding an action that one is interested in within a larger task probably makes participants perform that action in a manner that is closer to how they do so in daily life.

When asking participants to perform a task, we are not only informing them about what they are required to do but also indirectly creating expectations about the relevance of various components of the task. We show that this has an impact on people’s gaze. Thus, where people look is not only a function of the task and environment. This should be considered when making inferences about behavior in daily life on the basis of experiments, even if the experiments were conducted in a natural setting. In daily life, people are seldom instructed to do specific tasks, but they perform tasks spontaneously to satisfy their own needs (preparing a cup of tea) or to reach certain destinations (climbing a flight of stairs). The outcome of the current study suggests that experimenters should consider embedding the task of interest within a realistic context if the goal is to gain knowledge about behavior in daily life.

### Limitations of the study

We did not accompany the participants while they performed the experimental task because it is known that the presence of other people can influence gaze,^10^^,^^12^^,^^13^^,^^41^ but we had to tell them that we were measuring their eye movements, because they had to allow us to do so, and they were of course aware of wearing the eye tracker. We may have found less effect of the way we presented the task if participants had been unaware that we were measuring their eye movements because being more aware of where they look is likely to make their gaze more susceptible to biases.^42^^,^^43^

There are obvious benefits of studying behavior in a natural setting, but there are also challenges. Having some variability in how the task is presented and experienced can help ensure that the outcome of the study is generalizable to slightly different circumstances. But there is a cost to abandoning well-controlled experimentation: it makes it much more difficult to identify the exact cause of any effect that is found, which is often the main goal of the study. Thus, some compromise has to be found. Despite our attempt to recreate a setting that is close to the one normally encountered in daily life, we excluded one participant who looked at her phone and another who met other people while navigating the staircase. These situations are common in daily life, but they are also likely to influence gaze just as task instructions do. Thus, introducing some constraints seems to be inevitable if one wants to test how a specific factor influences gaze without testing a huge number of participants under very many circumstances.

Finally, while the use of a calibration-free eye tracker should prevent systematic changes in gaze estimation over time, future studies should consider including an assessment of data quality. The accuracy and precision of the eye tracker should specifically be evaluated in the context of the critical part of the experiment (as we have done previously and found good precision in a comparable task; see Ghiani A. ^17^), as the accuracy and precision of eye tracking data are likely to be influenced by the nature of the specific task. Here, we relied on our assessment of data quality in our earlier study using the same equipment,^17^ but it would have been better to assess the quality again in precisely these circumstances.

## STAR★Method

### Key resources table

**Table:** 

REAGENT or RESOURCE	SOURCE	IDENTIFIER
**Deposited data**		

Database (video data are not included)	Open Science Framework	https://osf.io/45wbh/?view_only=34a3327dd80c4c1bad7f2b799087c33c

**Software and algorithms**		

Python	Python Software Foundation	
OpenCV	Bradski^44^	
Pingouin	Vallat^45^	

### Resource availability

#### Lead contact

Further information and requests for resources should be directed to the lead contact, Andrea Ghiani (a.ghiani@vu.nl).

#### Material availability

This study did not generate new unique reagents.

#### Data and code availability

• The analyzed data have been deposited at Open Science Framework (OSF) and are publicly available. The link is listed in the key resources table. Our ethical approval does not allow us to publish the raw video data.
• All original Python codes to visualize and analyze the data have been deposited at Open Science Framework (OSF) and are publicly available. The link is listed in the key resources table.
• Any additional information required to reanalyse the data reported in this paper is available from the lead contact upon reasonable request.

### Experimental model and study participant details

#### Participants

Thirty-four participants (21–32 years old; 14 females) with normal vision (sometimes after correction with contact lenses) and no difficulties walking took part in the experiment. The experiment was conducted in accordance with approval by The Scientific and Ethical Review Board of the Faculty of Behavior and Movement Sciences of the Vrije Universiteit Amsterdam (file VCWE-2021-035), which included all participants providing written informed consent.

### Method details

#### The eye tracker

The direction of gaze (at 200 Hz) and video footage of the scene from the participant’s viewpoint (at 30 Hz) were measured using Pupil Invisible eye-tracking glasses (Pupil Labs, GmbH). This is a calibration-free wearable eye tracker (a description of their performance can be found at: arxiv:2009.00508). One advantage of a calibration-free eye tracker is there should be no systematic changes over time in gaze estimation due to drifts. Our estimate of their accuracy (variability in systematic errors) when measuring gaze while walking on staircases is that it is better than 0.5° in the lateral direction, almost 1.5° in the vertical direction when walking up the staircase, and almost 3° in the vertical direction when walking down the staircase. The precision (standard deviation across measurements) is about 1° in all cases.^17^ The systematic vertical errors when descending the staircase are probably larger as a result of gaze being directed far downwards, near the lower edge of the image.

#### Design and procedure

Participants were divided into two groups, a *Stairs-relevant* group (n = 18) and a S*tairs-irrelevant* group (n = 16; two planned participants did not show up). Both groups walked down and then up the same staircase. A trial started with the participant putting on the eye tracker and standing in front of the closed door of an apartment on the second floor of an apartment building. Once the recording started, participants placed in their pocket the phone onto which all the data were to be recorded. The experimenter then opened the door and the participant began walking. The *Stairs-relevant* group was instructed to walk down the flight of indoor stairs located just outside the door, and then, after reaching the entrance door of the building, to walk back up the staircase to return to the starting point. The S*tairs-irrelevant* group was instructed to walk to a square located outside the building and to look at a group of statues in that square. They were asked to look at the statues for 2 or 3 minutes before returning to the starting point. This task required participants to walk down the same stairs, to leave the building and walk for about 5 minutes to reach the square, and then to return to the building and walk back up the same stairs (total duration ∼12 mins). Figure 1 shows a schematic illustration of the task for the two groups. Crucially, both groups walked down and then back up the same staircase, but the *Stairs-relevant* group was explicitly instructed to do so, while for the *Stairs-irrelevant* group the stairs were just the initial (descending) and final (ascending) portions of their walk to the square. The staircase led from the second floor to the ground floor via one short staircase (4 steps), then one long staircase (12 steps), two short staircases (3 steps each) and one long staircase (9 steps). Only gaze when walking up and down the two long staircases was considered in the analyses. All steps had a run of 30 cm and a rise of 16 cm, leading to a staircase steepness of about 28°. Participants in the *Stairs-irrelevant* group were asked at the end of the experiment what they thought we were studying. Their answers were all related to the observation of the statues, with no mention of stair climbing. The experimenter did not follow or observe the participants as they walked around. Both groups were aware that their eye movements were being recorded.

#### Data analysis

After localizing the sections of the scene videos that contained the relevant staircases, gaze during those periods was subject to manual coding to label each fixated structure starting from the first fixation on any step and ending after the last fixation on any step. Videos were inspected using the open-sourced python library OpenCV.^44^ The coder (first author) was not blinded to the condition for each participant. A step was considered to have been fixated if gaze was judged to be directed towards the same part of the step for at least two frames (about 66 ms). Sequential fixations on the same step were merged under the same step number. Likewise, multiple fixations made on non-steps were merged under the same label “elsewhere” (as in reference 17). For each participant, we obtained a sequence of fixated steps, interleaved by periods of looking elsewhere. In order to provide a general description of the gaze sequence during stair climbing, we examined how gaze transitioned between steps across successive fixations by computing the number of steps between pairs of successively fixated steps (*Direct* distribution in Figure 3). The sign of this difference indicates the direction of the next fixation: shifting gaze to a step that will be reached later was positive and shifting gaze to a step that will be reached earlier was negative. Transitions were treated separately if gaze shifted elsewhere before shifting back to one of the steps (*Indirect* distribution in Figure 3). In that case, it was possible that there could be *no steps* between pairs of successively fixated steps: gaze could shift away from a step and then back to the same step.

The fraction of steps that were fixated was obtained by dividing the number of distinct steps that were fixated in each staircase by the total number of steps in that staircase. To obtain one value for the descent and one for the ascent for each participant, we averaged the fraction of fixated steps across the two staircases. We also considered steps that were fixated before the participant had reached each staircase. However, we only considered gaze from 2 seconds before the participants stepped on the first step of each staircase, because especially when walking up the first staircase, the steps were already visible when participants were near the entrance to the building (see Start ascent in Figure 1). We ignored glances towards the staircase at that time because it was difficult to interpret which step a participant was looking at long before they reached the staircase, and presumably at that time they were not localizing the step to guide their later foot placement. The moment of the first step was determined from the output of the inertial measurement unit (IMU) in the eye tracker (that could be used to estimate the head’s vertical displacement). We considered the time of the head’s lowest position during each stride as the moment that the foot was placed stably (as in reference^17^). We also checked whether the total time spent on the staircase was different between the two groups. We computed the time between when the participant stepped onto the first step and the time when the last step was reached for each staircase. The total time spent on the staircase was the sum of the times for the two staircases for the participant in question. It was determined separately for descent and ascent.

In addition to examining where participants looked, we also analyzed the eye movements themselves. Fast eye movements that shift gaze (saccades) were identified in the eye movement data using a custom-built script written in Python. After removing blinks detected by the Pupil Invisible blink detection algorithm, saccades were recognized on the basis of the velocity at which gaze shifted, together with the consistency in the direction in which gaze shifted across samples. A section of the gaze recording was considered to belong to a saccade if the dot product of the velocity on consecutive samples was more than 10 times the median absolute value of the dot product of the velocity on consecutive samples during that session (we checked that these median values did not differ systematically between the groups). We only considered saccades with an amplitude of at least 3 degrees. Moreover, the interval between saccades had to be at least 100 ms (otherwise, the slower of the two was removed). For this analysis, we consider the time between when the participant first fixated any step starting from 2 seconds before reaching the first step and when the participant reached the last step. For this time interval, we computed the amplitudes and directions of all saccades, separately for both staircases. For each participant, we then determined the average saccade amplitude by averaging the saccade amplitudes across the two staircases. Additionally, we computed the frequency distribution of the angular directions of saccades of various sizes. Directions were grouped into 40 bins of 9 deg each.

### Quantification and statistical analysis

The statistical analyses were performed using Python (version 3.9.7) using the open-source statistical package Pingouin (version 0.3.5).^45^ A mixed model ANOVA with Direction as a within-subjects factor (Ascending *vs.* Descending) and Group as a between-subjects factor (Stairs relevant *vs.* Stairs irrelevant) was performed on three dependent variables: fraction of fixated steps, saccade amplitude and time on stairs. Statistical significance level was set at p < 0.05. One participant from the *Stairs-relevant* group was excluded from the analysis, both when ascending and descending, as he completed the task in a fundamentally different way to all other participants: he ran both up and down the staircases. He later mentioned doing so due to anxiety in performing the task. One participant from the *Stairs-irrelevant* group was excluded from the analysis, both when ascending and descending, because she used her phone (despite being asked not to do so) and took a facemask from her pocket while navigating the staircase. Finally, the data when descending were excluded from the analysis for one participant from the *Stairs-irrelevant* group because other people were present on the staircase, which might also have influenced the participant’s gaze. This left us with 17 participants in the *Stairs-relevant* group and 14 in the *Stairs-irrelevant* group when descending, and 17 participants in the *Stairs-relevant* group and 15 in the *Stairs-irrelevant* group when ascending.
